# Exergy Flow as a Unifying Physical Quantity in Applying Dissipative Lagrangian Fluid Mechanics to Integrated Energy Systems

**DOI:** 10.3390/e26090791

**Published:** 2024-09-14

**Authors:** Ke Xu, Yan Qi, Changlong Sun, Dengxin Ai, Jiaojiao Wang, Wenxue He, Fan Yang, Hechen Ren

**Affiliations:** 1 Electric Power Research Institute of State Grid, Tianjin Electric Power Company, Tianjin 300010, China; xudenke@yeah.net (K.X.); qiyan_fly@163.com (Y.Q.); adx1991@163.com (D.A.); 2 State Grid Smart Internet of Vehicle Co., Ltd., Beijing 100052, China; 3 Center for Joint Quantum Studies, Department of Physics, Tianjin University, Tianjin 300350, China; sunchanglong@tju.edu.cn (C.S.); wangjiaojiao@tju.edu.cn (J.W.); hewenxue@tju.edu.cn (W.H.); fanyangphys@tju.edu.cn (F.Y.); 4 Tianjin Key Laboratory of Low Dimensional Materials Physics and Preparing Technology, Department of Physics, Tianjin University, Tianjin 300350, China; 5 Joint School of National University of Singapore and Tianjin University, International Campus of Tianjin University, Binhai New City, Fuzhou 350207, China

**Keywords:** integrated energy systems, exergy flow, Lagrangian fluid mechanics

## Abstract

Highly integrated energy systems are on the rise due to increasing global demand. To capture the underlying physics of such interdisciplinary systems, we need a modern framework that unifies all forms of energy. Here, we apply modified Lagrangian mechanics to the description of multi-energy systems. Based on the minimum entropy production principle, we revisit fluid mechanics in the presence of both mechanical and thermal dissipations and propose using exergy flow as the unifying Lagrangian across different forms of energy. We illustrate our theoretical framework by modeling a one-dimensional system with coupled electricity and heat. We map the exergy loss rate in real space and obtain the total exergy changes. Under steady-state conditions, our theory agrees with the traditional formula but incorporates more physical considerations such as viscous dissipation. The integral form of our theory also allows us to go beyond steady-state calculations and visualize the local, time-dependent exergy flow density everywhere in the system. Expandable to a wide range of applications, our theoretical framework provides the basis for developing versatile models in integrated energy systems.

## 1. Introduction

The worldwide revolution towards renewable energy sources has largely diversified our energy supply system while increasing its complexity at the same time. Power grids nowadays face the design challenge of integrating fossil fuel power, solar power, wind power, hydropower, and nuclear power all into one system and optimizing its efficiency. A universal quantity to relate and combine these very different sources is therefore in high demand. Energy seems to be the most natural currency when converting one form to another, given that energy is the resource we would like to harvest. Yet, very soon, the community realized energy comes in different grades in terms of the quality or usefulness of the energy contained in each physical form [1,2,3]. In other words, not all energies are equal when it comes to their readiness to be consumed by end users. Conventional designs convert most forms of energy into electricity and transfer it using electric power grids. However, electricity itself is not always the optimal form of energy to meet our ever-growing needs, which also include heating, cooling, and gas transport [4,5,6,7]. Hence, a true modern multi-energy system demands a holistic approach to analyzing the different forms of energy on a fundamental level. In this light, the field of unification theories for integrated energy systems has gained traction and is rapidly growing.

At the heart of a unification theory is one physical quantity that governs the physical dynamics in all energy forms, ideally one that emerges from the point of view of fundamental physics. So far, most endeavors to unify energy forms such as gas, fossil fuel, and electricity have been directed to the comparison of the mathematical formulae between the equations of motion. The popular approach includes writing down the systems’ governing differential equations and drawing parallels between them and those in an electric circuit [8,9,10,11,12,13,14,15]. This methodology has achieved successes in the area of efficient equation solving. However, its effort to unify the energy forms stops on a mathematical level and does not provide a holistic view from the perspective of physics. What the field of integrated energy systems still needs is a theoretical framework to incorporate diverse branches of physics and bring them into one playground. Furthermore, this unifying framework should be able to address the conversions and transfers from one energy form to another at various nodes of complex multi-energy power grids instead of calculating the lumped-sum energy of each component separately and merely juxtaposing them.

The physics community once faced a similar problem of incorporating the coexistence of multiple types of quantum fields including fermions and bosons with rich interactions among them. They tackled it using Lagrangian mechanics and developed quantum field theory [16,17]. Lagrangian mechanics is an alternative framework to formulate classical and quantum mechanics starting from the least action principle of a dynamic system. By examining the different terms of kinetic and potential energies all at once, it provides the benefits of predicting how a complex system evolves with time and allowing coupling terms between subsystems. These powerful features would render Lagrangian mechanics the perfect framework to describe integrated energy systems except for one fatal flaw: traditional Lagrangian mechanics focuses on conservative systems and does not address dissipation. On the other hand, gas, electricity, and heating systems are all fluidic in nature, where dissipation from friction and loss play critical roles in the energy efficiency, and energy is not conserved. If we wish to adopt the Lagrangian mechanical framework for the field of integrated energy systems, we need to modify it to be applicable to dissipative systems. Early works in the field of Lagrangian fluid mechanics start from the minimum entropy production principle and provide a foundation for us to build on [18,19,20,21,22,23,24].

Here, we adopt Lagrangian fluid mechanics in the presence of dissipation to describe integrated energy systems. Most importantly, we identify exergy flow as the unifying physical quantity across subsystems, replacing the concept of energy in traditional theories. This new paradigm allows us to develop a complete theory that incorporates the various energy forms to combine and couple them, which has not been achieved in the field of multi-energy systems. To illustrate the novelty of our approach, we apply this new theoretical framework in the simple case of a coupled heat–electricity system. Using the formula we develop in this work, we map the exergy loss rate at various locations in the system and compare our results to those calculated using traditional exergy analysis methods. Under steady-state conditions, they yield consistent results, although our method is not only applicable to steady-state but also to transient calculations. Moreover, our method includes exergy loss terms due to the mechanical energy transfer and viscosity of fluid, thereby greatly expanding the applicability of exergy analysis.

## 2. Dissipative Lagrangian Fluid Mechanics

We start with a brief review of Lagrangian mechanics. In dissipationless systems where energy is conserved, the Lagrangian of a mechanical system is defined as the total kinetic energy minus the total potential energy, i.e., $L=T_{k}-V$. Using the variational principle, Lagrangian mechanics distills a scalar quantity—the Lagrangian—as opposed to the vector forces, positions, and momenta in the Newtonian framework, and hence does not depend on specifics such as the choice of coordinates. The dynamics of a conservative system is then given by the least action principle, which states that physical variables such as position and momentum evolve with time in such a way that the total action—the integral of the Lagrangian—is minimized.

**Figure 1:**
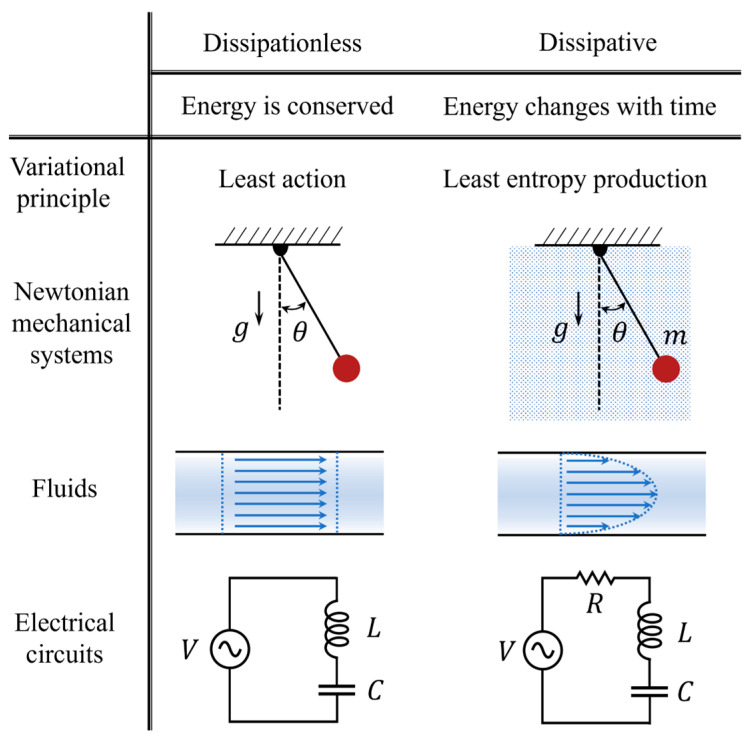
Comparison of Lagrangian mechanics between non-dissipative and dissipative systems.

Lagrangian mechanics can be widely applied to all physical systems such as Newtonian mechanical systems, ideal fluids, and electrical circuits [25,26], as illustrated in Figure 1.

At last, let us turn to fluid mechanics. Consider a fluid flowing through a pipe, water in an isothermal environment, for example. In the absence of viscosity and heat exchange, the system is dissipationless. Let ρ, v, and p be the density, flow velocity, and pressure, respectively. The equations of motion of the system are given by the continuity equation

(1) $$\frac{\partial \rho }{\partial t}+\nabla \cdot \left(\rho v\right)=0,$$

and Newton’s second law

(2) $$\rho \frac{dv}{dt}+\nabla p=f,$$

where f is the per volume density of any conservative external force such as gravity or the electrostatic force.

Defining the per volume gravitational potential energy as Φ, we can write the Lagrangian of an infinitesimal volume of fluid as

(3) $$L=T_{k}-V=\frac{1}{2}\rho v^{2}\delta V-p\delta V-\Phi \delta V,$$

where $T_{k}=\frac{1}{2}\rho v^{2}\delta V$ is the kinetic energy, pδV is the internal energy, and ΦδV is the potential energy corresponding to the external force with f=−∇Φ. The Euler–Lagrange equation dictates $\frac{d}{dt}\frac{\partial L}{\partial \dot{q}}-\frac{\partial L}{\partial q}=0$. Taken the generalized coordinates q to be $x_{i}\;\left(i=1,\;2,\;3\right)$ and generalized velocities $\dot{q}$ to be $v_{i}\;\left(i=1,\;2,\;3\right)$, we obtain

(4) $$\left(\frac{d}{dt}\frac{\partial }{\partial v_{i}}-\frac{\partial }{\partial x_{i}\;}\right)\frac{1}{2}\rho v^{2}=\rho \frac{dv_{i}}{dt},$$

(5) $$\left(\frac{d}{dt}\frac{\partial }{\partial v_{i}}-\frac{\partial }{\partial x_{i}\;}\right)p=-\frac{\partial p}{\partial x_{i}\;},$$

(6) $$\left(\frac{d}{dt}\frac{\partial }{\partial v_{i}}-\frac{\partial }{\partial x_{i}\;}\right)\Phi =-\frac{\partial \Phi }{\partial x_{i}\;}=f_{i}.$$

Putting these terms together would thus give back the individual components of our vector equation of motion:

(7) $$\rho \frac{dv_{i}}{dt}+\frac{\partial p}{\partial x_{i}\;}-f_{i}=0.$$

In realistic scenarios, fluids dissipate energy both through viscosity and through heat transfer. Since energy is not conserved, the least action principle no longer applies, and one must reconsider the variational principle that governs the system.

Dating back to Helmholtz in 1869 and Rayleigh in 1913, the minimum entropy production principle has been proposed, challenged, and advanced in the historical development of a theory for dissipative fluids using variational methods [27]. That the rate of entropy production in a viscous fluid obtains its minimum was first established under restricted conditions such as when the motion is irrotational and the forces and the fluxes are varied separately [18,19,28,29,30]. The field has since expanded the application of the minimum entropy principle to broader scenarios [20,21,22,23,31,32]. In the later development of the theory, the resulting Lagrangian integrand in the variational method, which equals the rate of exergy change per volume, or exergy flow density, has taken on comprehensive forms that include the kinetic and thermodynamic components for both reversible and irreversible processes, which we will readily adopt below for our calculations.

The mechanical component of the Lagrangian integrand, after including viscosity-related dissipation, is given by [23,28]

(8) $${\dot{e}}_{m}=-\rho v\cdot \frac{dv}{dt}-v\cdot \nabla p+v\cdot f{-\eta D}_{visc},$$

where η is the fluid’s viscosity in units of $\mathrm{kg}/\left(m\cdot s\right)$ and

(9) $$D_{visc}=\sum_{i,j}{\frac{1}{2}\left(\frac{\partial v_{i}}{\partial x_{j}}+\frac{\partial v_{j}}{\partial x_{i}}\right)}^{2}$$

is the viscous dissipation function.

We will refer to ${\dot{e}}_{m}$ as the mechanical exergy flow density, which represents the rate of change in the maximum possible work a system could do from the mechanical part of its energy. The first term $-\rho v\cdot \frac{dv}{dt}\;$ represents the rate at which kinetic energy is carried out of the unit volume, which decreases exergy, and hence carries a negative sign. The second term −v⋅∇p represents the rate at which work is done to the surrounding fluid in the form of pressure gradient, also decreasing exergy. The third term v⋅f represents the rate at which work is performed by external forces, which increases exergy, and hence carries a plus sign. The last term ${-\eta D}_{visc}$ represents the rate at which energy dissipates due to viscosity, decreasing exergy again.

It is noteworthy that the definition of ${\dot{e}}_{m}$ in Equation (8) is opposite in sign to that in Sciubba’s original work (see Equation (4) in Ref. [23]). According to Sciubba’s definition and the minimum entropy production principle, the exergy destruction should be minimized, whereas the expression we defined in Equation (8) corresponds to the exergy production rate, which would be maximized. This reversal in sign causes no influence on the resulting Euler–Lagrangian equations but helps make the physical meaning of the Lagrangian more intuitive.

In the presence of heat dissipation to the environment, we should also consider the thermal part of the exergy flow

(10) $${\dot{e}}_{t}=-(T-T_{0}){\dot{s}}_{irr},$$

where T and $T_{0}$ are the temperature of the fluid and environment while ${\dot{s}}_{irr}$ denotes the rate of change of entropy per unit volume due to heat dissipation. Hence, the total exergy flow density would be

(11) $$\dot{e}={\dot{e}}_{m}+{\dot{e}}_{t}.$$

If we replace the original definition of L [Equation (3)] with this expression of exergy flow density $\dot{e}$ (i.e., let $L\equiv \dot{e}$), under the minimum entropy production principle, the new Euler–Lagrange equation reads [23]

(12) $$\frac{\partial L}{\partial v_{k}}-\sum_{j}\frac{\partial }{\partial x_{j}}\left(\frac{\partial L}{\partial \left(\frac{\partial v_{k}}{\partial x_{j}}\right)}\right)=0.$$

Considering that $D_{visc}$ and ${\dot{e}}_{t}$ are not explicitly dependent of v, the first term gives

(13) $$\frac{\partial L}{\partial v_{k}}={\left(\rho \frac{dv}{dt}+\nabla p-f\right)}_{k}.$$

Meanwhile, with $\frac{\partial v_{j}}{\partial x_{j}}=0$ for incompressible fluids, the second is simplified to

(14) $$\sum_{j}\frac{\partial }{\partial x_{j}}\left(\frac{\partial L}{\partial \left(\frac{\partial v_{k}}{\partial x_{j}}\right)}\right)=-\sum_{j}\eta \frac{\partial }{\partial x_{j}}\left(\frac{\partial D_{visc}}{\partial \left(\frac{\partial v_{k}}{\partial x_{j}}\right)}\right)=-\sum_{j}\eta \frac{\partial^{2}v_{k}}{\partial x_{j}\partial x_{j}}=-{\left(\nabla^{2}v\right)}_{k}.$$

Combining these two terms turns the Euler–Lagrange equation into

(15) $$\frac{dv}{dt}+\frac{\nabla p}{\rho }-\frac{f}{\rho }-\mu \nabla^{2}v=0.$$

Here, μ=η/ρ is the kinematic viscosity in units of $m/s^{2}$.

This is the familiar Navier–Stokes equation, which governs the motion of a viscous fluid. This result verifies our using the expression $\dot{e}$ in Equations (8), (10), and (11) as the Lagrangian for viscous fluid dynamics where the system is dissipative.

## 3. Application to Coupled Heat–Electricity Systems

In the derivation above, we handle the dissipative nature of a fluid mechanical system by changing the difference between kinetic energy and potential energy to the rate of change in exergy. This modification allows us to inherit the framework of Lagrangian mechanics, at the price of changing both the physics and units of the Lagrangian itself. 

Next, we apply our theory based on exergy flow to a system with combined thermal and electric power. With the temperature of the environment considered, we derive the full expression of the exergy flow of the system. After that, we calculate the distribution of exergy flow density. Finally, we compare our results with those obtained from traditional methods of exergy analysis to verify the reliability and superiority of our theory.

### 3.1. Model Description

**Figure 2:**
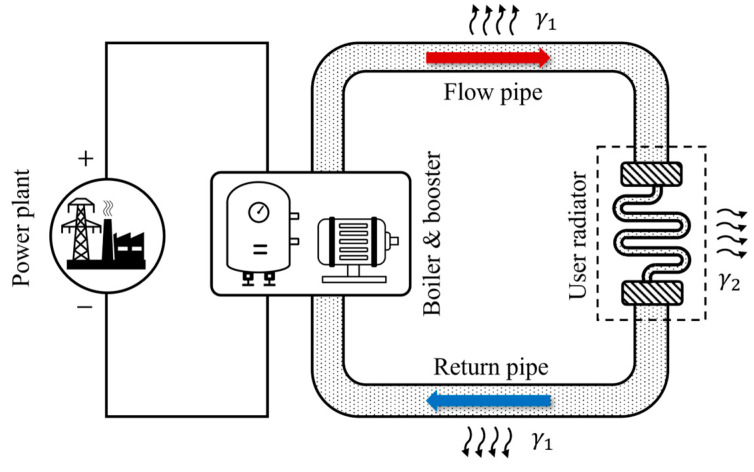
Schematic diagram of the coupled heat–electricity system discussed in the main text. The system consists of a power plant, electric boiler, booster pump, flow pipe (supply pipeline), return pipe, and heat load (user radiator). The parameters of all components are described in the main text.

We consider a coupled heat–electricity system for heating. The system consists of a power plant, an electric boiler, a booster pump, a user radiator, and two water pipelines, as illustrated in Figure 2. The heating system uses water as the working fluid. The direction of the water flow is indicated by the arrows in Figure 2. The power plant provides electricity to the boiler and booster pump. The boiler heats up the water, while the booster pump maintains the flow rate of water in the pipelines. The user radiator acts as the heat load. The temperature at the beginning of the flow pipe is controlled by the heating power of the boiler.

We assume that the flow and return pipelines both have a length of $L_{1}=5\;$ km, a heat dissipation coefficient of $\gamma_{1}=2.8\;$ W/(K∙m), and a diameter of $D_{1}=0.252\;$ m. The water flow rate is fixed at G=100  kg/s, corresponding to a flow speed of $v_{1}=2\;$ m/s.

The user radiator is modeled as a pipeline with a large heat dissipation coefficient of $\gamma_{2}=2000\;$ W/(K∙m). Its length and effective diameter are assumed to be $L_{2}=100\;$ m and $D_{2}=0.113\;$ m. At G=100  kg/s, the flow speed in the user radiator is $v_{2}=10\;$ m/s. We assume that the pipelines are horizontally placed, and thus the effect of gravity on the water flow is neglected.

Using the parameters above, the time-independent electrical power for maintaining the water flow rate is calculated to be 10.05 kW (see Section 3.2 for details).

The ambient temperatures of the flow and return pipes are both set to $T_{0}=0$ °C, while the ambient temperature of the user radiator is set to $T_{0,user}=20$ °C. For simplicity, the viscosity and density of water are assumed to be temperature independent. They take fixed values of $\eta =0.008\mathrm{kg}/\left(m\cdot s\right)$. and $\rho =1\times 10^{3}\mathrm{kg}/m^{3}$ all through our calculation. 

### 3.2. Mechanical Exergy Flow of Long Cylindrical Pipes

For water flowing in a long cylindrical pipe, in steady states, the expression of the mechanical exergy flow ${\dot{e}}_{m}$ [Equation (8)] can be further simplified by transforming it into a function of $\bar{v}$. Here, the average flow speed $\bar{v}$ is defined as $\bar{v}=G/(A\rho )$, where G and A are the water flow rate and the cross-sectional area of the pipe, respectively.

Consider a long cylindrical pipe with a radius R in zero external field. Let x and r be the coordinates in the axial and radial directions of the pipe, respectively. In steady states (i.e., $\frac{dv}{dt}=0$), the velocity v has only a non-zero component in the x direction and depends only on r, i.e., $v={(v}_{x}\left(r\right),0,0)$. Meanwhile, the pressure p depends only on x, i.e., p=p(x). In this case, both the first and third terms in Equation (8) are zero, resulting in

(16) $${\dot{e}}_{m}=-v\cdot \nabla p{-\eta D}_{visc}.$$

For convenience of discussion, hereafter we denote the pressure-related term −v⋅∇p and the viscosity-related term ${-\eta D}_{visc}$ in Equation (16) as ${\dot{e}}_{p}$ and ${\dot{e}}_{visc}$, respectively.

Similarly, in a steady state with zero external field, the Navier–Stokes equation [Equation (15)] becomes

(17) $$\frac{\nabla p}{\rho }-\mu \nabla^{2}v=0.$$

Letting $k=\frac{\partial p}{\partial x}$ be the gradient of pressure along the direction of water flow, considering that $v={(v}_{x}\left(r\right),0,0)$, p=p(x), and μ=η/ρ, Equation (17) is then simplified to

(18) $$k=\frac{\partial p}{\partial x}=\eta \frac{1}{r}\frac{\partial }{\partial r}\left(r\frac{\partial v_{x}}{\partial r}\right).$$

Solving Equation (18) gives the distribution of $v_{x}$ in the radial direction:

(19) $$v_{x}(r)=-\frac{k}{4\eta }\left(R^{2}-r^{2}\right).$$

With Equation (19), the average flow speed $\bar{v}$ is calculated to be

(20) $$\bar{v}=\frac{1}{\pi R^{2}}\int_{0}^{R}dr2\pi rv_{x}\left(r\right)=-k\frac{R^{2}}{8\eta },\;$$

which gives

(21) $$k=\frac{\partial p}{\partial x}=-\frac{8\eta }{R^{2}}\bar{v}.\;$$

Substituting Equation (21) into Equation (19), we obtain

(22) $$v_{x}=2\bar{v}\left(1-\frac{r^{2}}{R^{2}}\right).$$

which reflects the laminar flow velocity distribution in a cylindrical pipe.

Next, we use Equations (21) and (22) to evaluate the two terms in Equation (16). The pressure-related term ${\dot{e}}_{p}$ becomes

(23) $${\dot{e}}_{p}=-v\cdot \nabla p=2\left(1-\frac{r^{2}}{R^{2}}\right)\frac{8\eta {\bar{v}}^{2}}{R^{2}},$$

where ${\dot{e}}_{p}$ is positive because in this case, work is done to water to maintain the pressure for a steady flow rate. By averaging over the cross-section of the pipe, ${\dot{e}}_{p}$ is further simplified to

(24) $${\dot{e}}_{p}=\frac{1}{\pi R^{2}}\int_{0}^{R}2\pi r\left[2\left(1-\frac{r^{2}}{R^{2}}\right)\frac{8\eta {\bar{v}}^{2}}{R^{2}}\right]dr=\frac{8\eta {\bar{v}}^{2}}{R^{2}}.$$

Similarly, by averaging over the cross-section, the viscosity term

(25) $${\dot{e}}_{visc}={-\eta D}_{visc}=-\eta {\left(\frac{\partial v_{x}}{\partial r}\right)}^{2}=-\eta \frac{16{\bar{v}}^{2}r^{2}}{R^{4}}$$

becomes

(26) $${\dot{e}}_{visc}=-\frac{1}{\pi R^{2}}\int_{0}^{R}2\pi r\left(\eta \frac{16{\bar{v}}^{2}r^{2}}{R^{4}}\right)dr=-\frac{8\eta {\bar{v}}^{2}}{R^{2}}.$$

Combining Equations (24) and (26) leads to ${\dot{e}}_{visc}=-{\dot{e}}_{p}$ and

(27) $${\dot{e}}_{m}={\dot{e}}_{p}+{\dot{e}}_{visc}=0.$$

Equation (27) represents the local balance of exergy input and loss in the system under steady-state conditions. 

The exergy flow ${\dot{E}}_{p}=\int {\dot{e}}_{p}dV$ and ${\dot{E}}_{visc}=\int {\dot{e}}_{visc}dV$ can be calculated separately by integrating Equations (24) and (26) over the volume of the pipeline. In our system, $E_{p}$ represents the total exergy flow injected from the booster pump to maintain the water flow, while $E_{visc}$ is the total exergy flow due to the dissipation of viscosity. With the parameters given in Section 3.1, we obtain ${\dot{E}}_{p}=10.05\;$ kW, ${\dot{E}}_{visc}=-10.05\;$ kW, and ${\dot{E}}_{m}=0$, indicating that all exergy injected from the booster pump is just annihilated by the viscosity.

### 3.3. Thermal Exergy Flow of Long Cylindrical Pipes

Heat transfer to the environment leads to the destruction of exergy, giving rise to a negative exergy flow in the system. In the following, we calculate the thermal part of the exergy flow density ${\dot{e}}_{t}$ of a long cylindrical pipe with a cross-sectional area A and a heat dissipation coefficient γ.

For a pipe element of length dx, the power density of heat dissipation to the environment is given by

(28) $$\dot{q}=\frac{1}{A}\frac{d\dot{Q}}{dx}=\frac{\gamma }{A}\left(T-T_{0}\right),$$

where T and $T_{0}$ are the temperatures of the fluid and environment, respectively. 

The per volume rate of entropy change due to heat dissipation is thus given by

(29) $${\dot{s}}_{irr}=\frac{\dot{q}}{T}=\frac{\gamma }{A}\left(1-\frac{T_{0}}{T}\right),$$

which corresponds to an exergy flow density

(30) $${\dot{e}}_{t}=-\left(T-T_{0}\right){\dot{s}}_{irr}=-\frac{1}{A}\cdot \frac{\gamma {\left(T-T_{0}\right)}^{2}}{T}.$$

For a given temperature distribution T(x), integrating Equation (30) over the volume of a pipe with length L gives the total thermal exergy flow of the system:

(31) $${\dot{E}}_{t}=\int {\dot{e}}_{t}dV=-\int_{0}^{L}\frac{\gamma {\left(T-T_{0}\right)}^{2}}{T}dx.$$

In the literature, a frequently cited formula for calculating the steady-state exergy flow of a cylindrical pipe is [1]

(32) $${\dot{E}}_{t}=cG\left[T_{2}-T_{1}+T_{0}\ln\left(\frac{T_{1}}{T_{2}}\right)\right],$$

where $T_{1}$ and $T_{2}$ are the fluid temperatures at the beginning and end of the pipe, and c and G denote the specific heat capacity and flow rate of the fluid, respectively. The derivation of Equation (32) is based on the following assumption: at a given time t, the heat dissipation of the pipe is equal to the difference in internal energy between the water flowing out of the pipe and the water flowing into the pipe at that time. Therefore, it only applies under steady-state conditions.

It is proven that (see Appendix A for details) Equations (31) and (32) are equivalent under steady-state conditions. However, Equation (32) is applicable only to steady-state conditions, whereas Equation (31) is suitable for both steady-state and transient-state calculations.

### 3.4. Distribution of Exergy Flow Density

Combining Equations (27) and (30) gives the total exergy flow density $\dot{e}$ and the density of exergy loss rate ${\dot{e}}_{loss}=-\dot{e}$ of a long cylindrical pipe:

(33) $$\dot{e}=-{\dot{e}}_{loss}={\dot{e}}_{m}+{\dot{e}}_{t}=-\frac{1}{A}\cdot \frac{\gamma {\left(T-T_{0}\right)}^{2}}{T}.$$

Next, we apply Equation (33) to the coupled heat–electricity system described in Section 3.1 and calculate the distribution of the exergy loss rate of the system.

**Figure 3:**
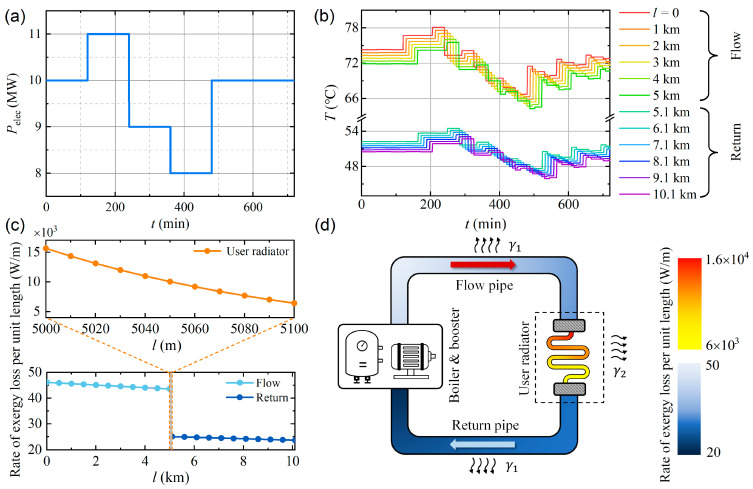
(**a**) Variation of electric power of the boiler over time. (**b**) Pipeline temperature versus time at various positions. (**c**) Rate of exergy loss per unit length at different positions. (**d**) Schematics of distribution of exergy loss rate per unit length of the system. The color on the left graph is schematic and is not strictly proportional to the color axis.

The power of the electric boiler is assumed to vary with time, as shown in Figure 3a. During the first two hours, the power of the boiler is maintained at 10 MW, and the system is in a steady state. At t= 120 min, the power is increased to 11 MW and holds for two hours. At t= 240 min, the power is decreased to 9 MW and holds for two hours. After that, the boiler power is further decreased to 8 MW and holds for another 2 h. Finally, the heating power of the boiler is increased to its initial value 10 MW at t= 480 min.

The water temperatures at different positions of the system are numerically calculated as a function of time using the finite element method. Figure 3b shows a set of T(t) curves with an interval of position Δl=1  km. At a given heat dissipation coefficient γ, the heat dissipation power $\gamma \left(T-T_{0}\right)$ decreases with decreasing T and therefore decreases with increasing l. Consequently, the temperature gradient dT/dl decreases as l increases, as illustrated by the decrement of the vertical spacing between the curves in Figure 3b.

The distribution of the per-unit-length exergy loss rate of the system is obatined by substituting the T(x) data into Equation (33), as plotted in Figure 3c. To present the distribution of the exergy loss rate in a more intuitive way, the exergy loss rate per unit length of the system is plotted as a color map shown in Figure 3d.

For our system, the rate of exergy loss is determined by the power of heat dissipation. Therefore, it is not surprising that the rates of exergy loss in the user radiator are three orders of magnitude higher than those in the flow and return pipes.

### 3.5. Comparison to Traditional Exergy Analysis Method

**Figure 4:**
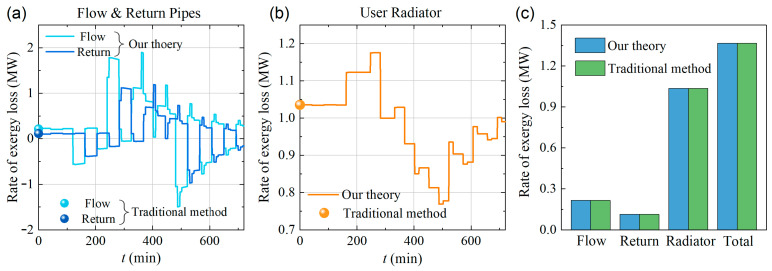
(**a**) Rate of exergy loss of the flow and return pipes. (**b**) Rate of exergy loss of the user radiator. (**c**) Exergy flow of different parts of the system at t=0, comparing our theoretical model with the traditional method.

Compared to the traditional exergy analysis method, our calculation based on Equation (33) has two main advantages. Firstly, it is applicable not only to the systems in steady states but also to those in transient states. Secondly, it considers the exergy loss terms from both mechanical energy transfer and heat dissipation. To demonstrate that our theory is consistent with the traditional exergy analysis methods, we compared the results obtained from both approaches, as shown in Figure 4. 

The solid lines in Figure 4a,b illustrate the time dependence of the exergy loss rate calculated using Equation (33), demonstrating the applicability of our theory to transient-state calculations. The color balls at t=0 in Figure 4a,b represent the results obtained using the traditional method [Equation (32)], showing perfect consistency with those calculated using our theory. 

A full comparison of the steady-state results obtained at t=0 using two different methods is presented in the bar chart in Figure 4c. The exergy loss rates calculated using the two methods are identical, again validating the reliability of our theory.

## 4. Conclusions

Even though there has been a growing trend to use the concept of exergy in optimizing the efficiency of multi-energy systems, the existing works discuss exergy as an evaluated result of energy and often follow the approach of obtaining exergy from energy calculations. In this work, we take a more fundamental approach starting from the point of view of Lagrangian mechanics. In revisiting the theoretical method in the ideal fluid scenario, we motivate the need for a modified theory to describe dissipative integrated energy systems. Our theoretical discussions originate from the minimum entropy production principle, and we showed that the Euler–Lagrange equation derived from the exergy flow Lagrangian agrees with the Navier–Stokes equations for a viscous fluid. To demonstrate how the Lagrangian approach readily handles multiple energy forms, we further expand the Lagrangian to incorporate both the mechanical and thermal parts of the exergy flow.

We identify exergy flow as the unifying quantity across different forms of energy and demonstrate its usage in a simple heat–electricity model. Based on our derived formula, we calculate the real-space map of the exergy loss rate of the system, which is a useful tool for analyzing and visualizing modern energy grids. Integrating over the one-dimensional system, we compare our result of the total exergy flow to that derived from the conventional approach. Under steady-state conditions, our theory agrees perfectly with the traditional formula. The mechanical components of the calculation showcase our theory’s ability to incorporate more physics inside one unified Lagrangian.

Unlike the traditional formula, our theory is not limited to steady-state calculations but applies to a wider class of situations where the system possesses explicit time dependences, as is often the case in realistic scenarios. The one-dimensional model proposed in this work serves as an elemental building block to describing larger, more integrated energy networks with complex pipelines, exchangers, and converters. Overall, our theoretical framework provides the basis for developing complex theories in coupled multi-energy systems, opening new directions for exciting future work.

## Data Availability

The data presented in this study are available on request from the corresponding author.

## Nomenclature

**Symbol**	**Description**
A	cross-sectional area of the pipe
*c*	specific heat capacity
$D_{1}$, $D_{2}$	pipe diameters
$D_{visc}$	viscous dissipation function
$\dot{e}$, ${\dot{e}}_{m}$, ${\dot{e}}_{t}$, ${\dot{e}}_{p}$ and ${\dot{e}}_{visc}$	density of total, mechanical, thermal, pressure-related and viscosity-related exergy flow
${\dot{e}}_{loss}$	density of exergy loss rate
${\dot{E}}_{m}$, ${\dot{E}}_{t}$, ${\dot{E}}_{p}$, and ${\dot{E}}_{visc}$	mechanical, thermal, pressure-related and viscosity-related exergy flow
G	flow rate of fluid
f	per volume density of conservative external force
k	pressure gradient
$L_{1}$, $L_{2}$	pipe lengths
${\dot{s}}_{irr}$	rate of change of entropy per unit volume
T	temperature
$T_{0}$	temperature of the environment
$T_{0,user}$	ambient temperature of the user radiator
p	pressure
v	flow speed
$\bar{v}$	average flow velocity
L	Lagrangian
$T_{k}$	total kinetic energy
V	total potential energy
ρ	density
Φ	per volume gravitational potential energy
η	viscosity
μ	kinematic viscosity
$\gamma_{1}$, $\gamma_{2}$	heat dissipation coefficient

## Appendix A. Equivalence of Equations (31) and (32) in Steady States

Under steady-state conditions, Equations (31) and (32) are equivalent. The proof is presented as follows.

For a pipe element with a length dx and a heat dissipation coefficient γ, the flows of heat into and out of the pipe element are given as cGT and cG(T+dT). Here, c and G are the specific heat capacity and flow rate of the fluid, respectively. From the conservation of energy, we obtain

(A1) $$cGT-cG\left(T+dT\right)=\gamma \left(T-T_{0}\right)dx.$$

Under steady-state conditions, T(x) is independent of time. Therefore, Equation (A1) can be transformed into

(A2) $$\frac{dx}{dT}=-\frac{cG}{\gamma \left(T-T_{0}\right)}.$$

Substituting Equation (A2) into Equation (31), we obtain

(A3) $${\dot{E}}_{t}=-\int_{0}^{L}\frac{\gamma {\left(T-T_{0}\right)}^{2}}{T}dx=cG\int_{T_{1}}^{T_{2}}\frac{T-T_{0}}{T}dT=cG\left[T_{2}-T_{1}+T_{0}\ln\left(\frac{T_{1}}{T_{2}}\right)\right],$$

which is exactly Equation (32).

Thus, the equivalence of Equations (31) and (32) under steady-state conditions has been proven.
